# Topical Delivery of Meloxicam using Liposome and Microemulsion Formulation Approaches

**DOI:** 10.3390/pharmaceutics12030282

**Published:** 2020-03-21

**Authors:** Julia Zhang, Anna Froelich, Bozena Michniak-Kohn

**Affiliations:** 1 Center for Dermal Research and Ernest Mario School of Pharmacy, Rutgers, The State University of New Jersey, 145 Bevier Road, Piscataway, NJ 08854, USA; julia.zhang@rutgers.edu; 2 Chair and Department of Pharmaceutical Technology, Poznan University of Medical Sciences, Grunwaldzka 6, 60-780 Poznań, Poland; froelich@ump.edu.pl

**Keywords:** liposome, transfersome, microemulsion, meloxicam, ex vivo skin permeation

## Abstract

The aim of this study is to develop, characterize and compare conventional liposome, deformable liposome (transfersome) and microemulsion formulations as potential topical delivery systems for meloxicam. Liposomes were characterized in terms of vesicle size, zeta potential and entrapment efficiency. For microemulsions, particle size, electrical conductivity and viscosity studies were performed to assess the structure of the investigated systems. An ex vivo skin permeation study has been conducted to compare these formulations. The dermal and transdermal delivery of meloxicam using these formulations can be a promising alternative to conventional oral delivery of non-steroidal anti-inflammatory drugs (NSAIDs) with enhanced local and systemic onset of action and reduced side effects.

## 1. Introduction

Meloxicam is a non-steroidal anti-inflammatory drug (NSAID) structurally related to the enolic acid class of 4-hydroxy-1,2-benzothiazine carboxamides. It was first approved as a 7.5 mg tablet (Mobic; Boehringer Ingelheim) by the United Stated Food and Drug Administration (US FDA) in 2000 and was later approved and marketed in capsule and suspension forms. These dosage forms are used clinically to treat acute and chronic pain and inflammation, as well as relieve swelling, stiffness and pain associated with arthritis. Additionally, meloxicam has been studied as a potential drug for Alzheimer’s disease and as a viable adjuvant therapeutic agent to treat different cancers, such as lung, colorectal, prostate and urinary bladder cancers [1,2,3,4,5]. However, adverse effects, such as gastro-intestinal toxicity/bleeding, headaches, rash, increased risk of cardiovascular events, etc., are frequently reported when this drug is administrated at high doses and with long-term treatment [6].

Topical administration provides a number of advantages over oral NSAIDs: the ability to deliver the drug substance more selectively to a specific site for both local and systemic effects, to avoid first pass effect, to reduce gastro-intestinal side effects and to improve patient compliance. As per European League Against Rheumatism (EULAR) and National Institute for Health and Care Excellence (NICE) guidelines [7], topical administrations of NSAIDs is recommended for the management of mild to moderate osteoarthritis pain before the oral route.

However, the barrier function of the skin impairs the penetration and absorption of drugs. Therefore, many formulation strategies, such as liposomes, nanoparticles, microemulsions, etc., have been assessed to overcome the barrier function of the *stratum corneum* (SC) and to improve drug transport into the skin.

Liposomes are spherical vesicles consisting of one or more phospholipid bilayers. Over the past 20 years, many studies have been conducted on liposomal delivery systems due to their biocompatibility, biodegradability, low toxicity and capability to encapsulate both hydrophilic and lipophilic drugs. However, the conventional liposome does not deeply penetrate skin but rather remains in the upper layer of the *stratum corneum* [8] due to its rigid structure and size [9].

Transfersomes^®^ belong to a class of highly elastic or deformable vesicles, which were first introduced by Cevc and Blume [10]. These are liquid-state vesicles that consist of phospholipids and an edge activator, which is often a single chain surfactant, e.g., sodium cholate, sorbitan esters (Span^®^ 60/65/80) and polysorbates (Tween^®^ 20/60/80), that destabilizes the lipid bilayers of the vesicles and increases their deformability by lowering the interfacial tension [11]. This feature enables the Transfersomes^®^ to squeeze themselves through intercellular regions of the *stratum corneum* under the influence of the transdermal water gradient. They have been reported to penetrate intact skin in vivo with an efficiency similar to that of subcutaneous administration, provided that the elastic vesicles are topically applied in non-occlusive conditions [10,12,13].

Microemulsions are thermodynamically stable liquid dispersions composed of polar and non-polar phases stabilized by one or more surfactants. One of the most important microemulsion features is an extremely low interfacial tension between the phases of different polarity. This is usually achieved with the use of a co-surfactant, an additional component revealing low molecular weight and good miscibility with both phases [14]. Another important property of microemulsions is small diameter of dispersed phase particles. It is noteworthy that numerous studies regarding topical and transdermal delivery of drugs incorporated in microemulsions indicate their significant potential as carriers that enhance absorption of the active ingredient [15,16,17,18,19,20]. Despite the fact that these systems have been known and investigated for more than 70 years [21], the exact mechanism explaining this phenomenon has not been elucidated. It has been hypothesized that several different factors might contribute to the increased topical absorption of the drug. One of them is the presence of surfactants and co-surfactants also acting as permeation enhancers and temporarily disrupting the organization of lipids in the *stratum corneum*. Another important feature of microemulsions is the small droplet diameter which may contribute to better penetration of the dispersed phase into the deeper skin layers. Moreover, in the case of oil in water (o/w) microemulsions, the oil phase might act as a drug reservoir, maintaining a high concentration gradient between the formulation and the skin [22].

In this study, we present both liposomal and microemulsion formulations investigated as potential carriers for the dermal delivery of meloxicam. In order to analyze the impact of structural features on the potential therapeutic efficacy, two different types of vesicles and two different microemulsion types were prepared and tested. Both liposomes and microemulsions were subjected to structural studies and applied to ex vivo skin in Franz diffusion cell studies.

## 2. Materials and Methods 

### 2.1. Materials

Soybean lecithin (SL) was purchased from Acros Organics (Morris Plains, NJ). Unsaturated Soybean Phosphatidylcholine (USPC) and Saturated Soybean Phosphatidylcholine (SSPC) were generously donated by LIPOID LLC (Newark, NJ, USA). Cholesterol (Chol) was purchased from Alfa Aesar (Haverhill, MA, USA). Cetylpyridinium chloride (CPC) was purchased from Sigma-Aldrich (St. Louis, MO, USA). Meloxicam (MX) was supplied from Acros Organics (Morris Plains, NJ, USA). Tween^®^ 85, triacetin, oleic acid, ethanol and isopropanol were purchased from Sigma-Aldrich. Transcutol^®^ P was kindly donated by Gattefosse (Paramus, NJ, USA). High-performance liquid chromatography (HPLC)-grade water and acetonitrile were purchased from Sigma-Aldrich and Midland Scientific (Omaha, NE, USA), respectively. Dermatomed human cadaver skin was obtained from New York Firefighter Skin Bank (New York, NY, USA). All other chemicals used were of reagent grade and purchased from VWR International (Radnor, PA, USA).

### 2.2. Methods

#### 2.2.1. Liposomes

##### Preparation

Meloxicam (MX)-loaded liposomes were prepared by the thin film hydration method followed by sonication [23]. Briefly, lipid mixtures of phosphatidylcholine (PC), cholesterol (Chol), MX and/or cetylpyridinium chloride (CPC), were dissolved in chloroform. The solvent was then evaporated under a nitrogen gas stream. The lipid film was placed in a desiccator for at least 12 h to remove any remaining solvent. The dried lipid film was hydrated with sodium acetate buffer solution (pH 5.5). Vesicles were subsequently sonicated in a sonicator bath (Tru-sweep Crest Bath Ultrasonicator, Cortland, NY, USA) for one hour followed by two cycles of 12 min probe sonication (SFX Branson Ultrasonic Processor, Emerson Industrial Automation, St. Louis, MO, USA) at continuous mode with 2-min intervals between the cycles in an ice-water bath. Liposome suspension was then centrifuged at 3000 g for 30 min. The prepared vesicle formulations (supernatant) were stored in airtight containers at 4 °C prior to use.

##### HPLC Method of the Quantification of MX

MX was quantified using high-pressure liquid chromatography (HPLC) with UV detection. The HPLC system included an Agilent 1100 Series liquid chromatograph (Agilent Technlogies, Santa Clara, CA, USA) and the Agilent Chemstation software (OpenLab CDS, ChemStation Edition, Rev. C.01.10, Agilent Technologies). A reversed-phase C18 column (YMC Triart C18 ExRS plus, 5 µm, 4.6 × 150 mm, YMC America Inc., Allentown, PA, USA) was used as the stationary phase. The column temperature was maintained at 30.0 ± 0.2 °C. The mobile phase composed of 1% phosphoric acid (A) and acetonitrile (B) at a flow rate of 1.0 mL/min. The gradient program is: 0 min, 35% B; 5.5 min, 75% B; 7.2 min, 35% B. The UV detector was set at a wavelength of 360 nm for MX. The retention time for MX was about 8.5 min. The method was linear at a concentration range of 0.05–50µg/mL with R^2^ of 0.9995 for meloxicam. The limit of detection (LOD) was found to be 0.05 µg/mL and the limit of quantification (LOQ) was 0.22 µg/mL. The relative standard deviation for both intra-day and inter-day precision was less than 2%.

##### Measurement of Vesicle Size, Size Distribution, Zeta Potential and Morphology

Average vesicle size and size distribution (Polydispersity Index, PDI) of the liposome formulations were measured by Dynamic Light Scattering (DLS) (Zetasizer Nano-S, Malvern Panalytical, Westborough, MA, USA). Zeta potential was measured by Electrophoretic Light Scattering (ELS) (Zetasizer Nano series, Malvern Panalytical). All formulation samples without further treatment were analyzed at room temperature.

The morphology of liposomes was characterized by transmission electron microscopy (TEM) (CM 12 TEM, Philips, Amsterdam, Netherlands). One drop of liposomal vesicle preparation was placed onto a copper grid, and the excess suspension was immediately adsorbed using filter paper. The sample was then stained by adding a drop of 2% phosphotungstic acid. The excess solution was immediately removed by filter paper, and then the sample was dried at room temperature. Afterward, the grid was observed using a TEM with AMT Image Capture Engine V602 (Advance, Microscopy Techniques Corp, Woburn, MA, USA).

##### Determination of MX Entrapment Efficiency

The concentration of MX in the vesicle formulation was determined by HPLC analysis after disruption of the vesicles with 50% *v/v* ethanol in water. The extracted solution was sonicated for 10 min in a sonicator bath (Tru-sweep Crest Bath Ultrasonicator, Cortland, NY, USA). The resulting solution was then filtered with a 0.45 µm nylon syringe filter (Midland Scientific, Omaha, NE, USA). The entrapment efficiency and drug loading of MX loaded in the liposomes were calculated according to Equations (1) and (2) [24], respectively.

% entrapment efficiency = (C_M_/C_i_) × 100 (1)

% drug loading = (C_M_/C_L_) × 100 (2)

where C_M_ is the concentration of MX loaded in the liposome, as described in the above methods, C_i_ is the initial concentration of MX added into the vesicle formulation and C_L_ is the concentration of phosphate lipid added into the vesicle formulation.

#### 2.2.2. Microemulsions

##### Pseudoternary Phase Diagrams

Pseudoternary phase diagrams were obtained with a water titration procedure [25]. The composition of the investigated systems is presented in Table 1. In the first step, the mixture of surfactant and co-surfactant (S_mix_) at a 1:1 ratio (*w/w*) was prepared. Next, the systems containing oil and S_mix_ at 1:9, 2:8, 3:7, 4:6, 5:5, 6:4, 7:3, 8:2 and 9:1 ratios (*w/w*) were obtained. The water phase was added to each sample dropwise, with gentle stirring during the titration process, until transparency loss was observed. Moreover, the viscosity of the system was visually inspected during the experiment. The transparent systems revealing low viscosity were classified as microemulsions. All experiments were performed at 25.0 ± 0.5 °C.

##### Electrical Conductivity Studies

**Figure 1:**
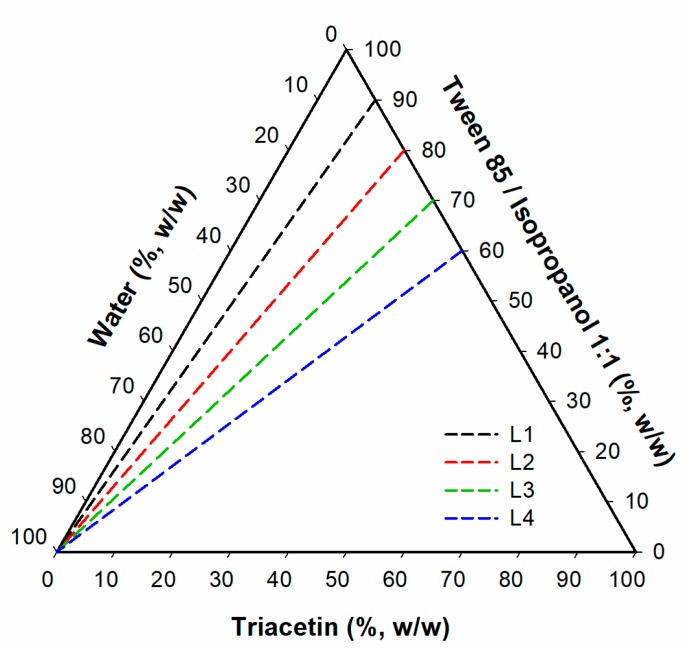
Dilution lines followed in conductivity studies. Lines L1, L2, L3 and L4 correspond to O:S_mix_ ratios 1:9, 2:8, 3:7 and 4:6 (*w/w*), respectively.

Electrical conductivity tests were performed with Thermo Orion model 105A+ (Thermo Fisher Scientific, Waltham, MA, USA) for the formulation 1B (Table 1). The device was calibrated with 12,896, 1413 and 100 μS cm^−1^ standard solutions. The conductivity studies were performed along the dilution lines L1, L2, L3 and L4 (Figure 1) corresponding to the initial mixture containing oil and S_mix_ at 1:9, 2:8, 3:7 and 4:6 ratios (*w/w*), respectively. Each sample was gradually diluted with 0.05% solution of sodium chloride and after the addition of each aliquot, the sample was gently mixed. All measurements were performed in triplicate at ambient temperature.

##### Viscosity Studies

The viscosity of microemulsion was monitored along the dilution lines depicted in Figure 1. The measurements were performed with Kinexus Ultra+ rotational rheometer (Malvern, UK) equipped with coaxial cylinders geometry (diameter: 25 mm, measurement gap: 4.2 mm). Each sample was analyzed in triplicate at 25.0 ± 0.2 °C. The shear rate was increasing linearly from 0 to 100 s^−1^ over 120 s.

##### DLS Studies

The particle size analysis was performed for selected microemulsions with Zetasizer Nano S equipped with a 632.8 nm He-Ne laser light source (4 mW). The measurements were done at 25.0 °C using non-invasive backscatter mode (NIBS) at an angle of 173°. Each sample was equilibrated prior the experiment for 180 s.

##### Preparation of Drug-Loaded Microemulsions

The composition of the microemulsions used in skin permeation experiments is presented in Table 2. In the first step, triacetin was mixed with surfactant and co-surfactant and MX (0.08% *w/w*) was dissolved in the resulting mixture. Next, water was added and the sample was gently mixed and inspected visually for clarity.

#### 2.2.3. Ex Vivo Skin Permeation Study

Cryopreserved full thickness dermatomed human cadaver skin derived from the posterior torso was obtained from New York Firefighters Skin Bank (New York, NY, USA). Upon receipt, the skin was kept frozen at −80 °C. On the day of study, the skin was quickly thawed in pH 7.4 PBS at room temperature for 20 min. An appropriate size of skin was cut and mounted between the donor and receptor chambers of a vertical Franz Diffusion Cell (FDC), the *stratum corneum* facing the donor chamber.

The receptor chamber was filled with a known volume of pH 7.4 PBS buffer and stirred continuously with a small PTFE-coated magnetic bar at 600 rpm. Temperature of the skin surface was maintained at 32 °C by placing the FDC into a dry block heater (Logan Instruments, Somerset, NJ, USA) set at 37 ± 0.5 °C.

After the assembled FDC is equilibrated for at least 30 min, 500 μL of each liposome and microemulsion formulation is applied to the skin. At appropriate time intervals, an aliquot of the receptor medium was withdrawn, and the same volume of fresh buffer solution was replaced to the receptor chamber. The concentration of MX in the aliquot was analyzed using the HPLC method described in Section 2.2.1.

At the end of the permeation study, the skin sample mounted on the receptor compartment was washed with PBS buffer (pH 7.4) to remove the residual formulations and dried with a cotton swab. Next, the part exposed to the test formulation was cut out with scissors and the dermal and epidermal layers were separated manually with tweezers. Separated layers were cut into small pieces and then homogenized with 50% ethanol. Homogenized skin samples were centrifuged at 10,000 rpm for 5 min and the supernatant was filtered into HPLC vials using 0.45 µm PTFE syringe filters. Collected samples were analyzed using the HPLC method described in Section 2.2.1.

#### 2.2.4. Statistical Analysis

The data were reported as mean ± standard deviation (SD) (*n* = 3). The obtained results were analyzed with one-way analysis of variance (ANOVA) followed with post-hoc Scheffé’s test. The statistical significance level in all tests was set at 5%. All calculations were performed with the use of Statistica 12.5 software (StatSoft Inc., Tulsa, OK, USA).

## 3. Results

### 3.1. Liposomes

#### 3.1.1. Selection of Phosphatidylcholine

As demonstrated in many published studies, liposome properties are considerably affected by the lipid type/composition, surface charge, size and the method of preparation. Furthermore, the choice of bilayer components determines the ‘rigidity’ or ‘fluidity’ and the charge of the vesicles. For instance, unsaturated phosphatidylcholine from natural sources (egg or soybean) yields much more permeable and less stable bilayers, while the saturated phospholipid with long acyl chains (for example, dipalmitoylphosphatidylcholine) forms rigid, rather impermeable vesicles [23,26,27,28].

The initial formulations were based on the MX-loaded menthosomes developed by Duangjit et al. [29]. The compositions of each formulation are listed in Table 3. The preparation procedure was as described in Section 2.2.1, but with only one cycle of 6 min probe sonication at continuous mode.

In this study, the type and grade of PC were evaluated. Specifically, soybean lecithin (SL), unsaturated phosphatidylcholine (USPC) and saturated soybean phosphatidylcholine (SSPC) were used for the preparation of liposome formulations at the concentration levels of 0.4, 0.8 and 1.2%.

**Figure 2:**
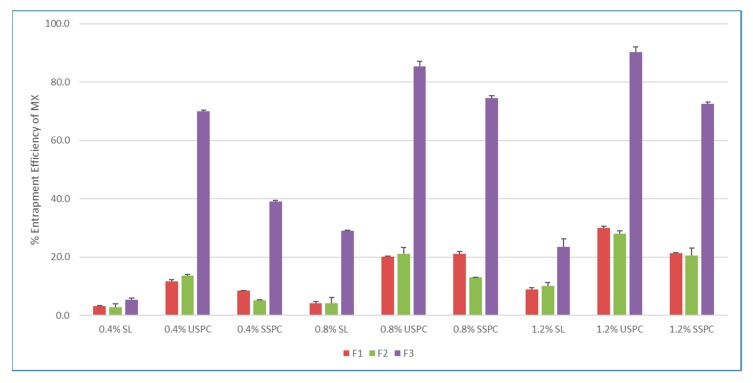
Effect of different grade of phospholipids (PL) on entrapment efficiency of meloxicam (MX). SL: soybean lecithin, USPC: unsaturated soybean phosphatydilcholine, SSPC: saturated soybean phosphatydilcholine. Bars are means ± standard deviation (SD), *n* = 3.

**Figure 3:**
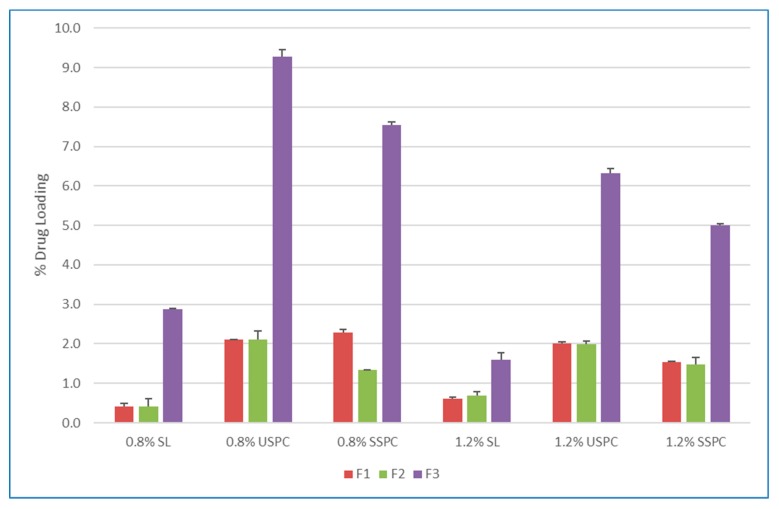
Effect of different grade of phospholipids (PL) on drug loading of meloxicam (MX). SL: soybean lecithin, USPC: unsaturated soybean phosphatydilcholine, SSPC: saturated soybean phosphatydilcholine. Bars are means ± SD, *n* = 3.

As Figure 2 demonstrates, compared to soybean lecithin, the entrapment efficiency improved dramatically with USPC and SSPC, in which USPC yielded the best results. The entrapment efficiency of transfersomes was significantly higher than that of the conventional liposomes. These results might be attributed to the intrinsic properties of the cationic surfactant as a solubilizer and the interactions among the surfactant, MX and lipid bilayer. The encapsulation rates increased with increased concentration of lipids, however, the drug loading decreased from 0.8% to 1.2%, as demonstrated in Figure 3.

The physical characteristics of the investigated formulations are described in Table 4.

As suggested in Table 4, the incorporation of different components in the liposome systems affected the size, zeta potential and size distribution of the vesicle formulation. Generally, liposomes prepared using USPC had smaller particle sizes than those prepared using SSPC but similar size with those prepared with SL. For example, at 0.8% concentration level, the particle sizes of F2 (conventional liposome with cholesterol) were found to be 125.7 ± 2.5 nm using SL, 125.7 ± 4.9 nm using USPC and 190.1 ± 1.4 nm using SSPC, respectively. A similar finding was observed for F3 (transfersome) at the same concentration level, the particle sizes for the vesicles prepared using SL, USPC and SSPC were 155.4 ± 11.2, 136.7 ± 5.7 and 199.0 ± 0.6 nm, respectively. The impact of the liposomes’ composition on the size was assessed because vesicular size has the ability to influence the penetration of drugs through the skin to the deeper layers. Verma et al. [30] studied the influence of liposomal size on the skin penetration utilizing two fluorescently labeled substances. They found that the penetration of these fluorescent substances was inversely related to the size of the liposomes. It was concluded that vesicles with a size larger than 600 nm failed to deliver the loaded molecules into deeper layers of the skin, whereas those with a size smaller than 300 nm were able to deliver the loaded molecules into the deeper layers of the skin. The size of the investigated vesicular systems using SL and USPC at all three concentration levels were less than 200 nm, which means that these investigated systems have the potential to deliver MX through the skin.

Polydispersity Index (PDI) had been measured to determine the degree of size distribution uniformity of these vesicle systems. In drug delivery applications using lipid-based carriers, such as liposomal formulations, a PDI of 0.3 and below is considered to be acceptable and indicates a homogenous population of phospholipid vesicles [31,32]. As elucidated in Table 4, the PDI of liposomal formulations prepared using SL and USPC was less than 0.3, with the only exception of F3 prepared using 1.2% USPC. However, the PDI of the vesicles prepared using SSPC were generally greater than 0.3, indicating that these nanoparticles are heterogeneously sized.

Zeta potential was used to study these vesicles’ surface charge, which was affected by the total net charge of the vesicle components and pH of the hydration buffer. The isoelectric point (pI) of MX is 2.6 [29], which is lower than the pH of hydration buffer (pH 5.5). Therefore, MX is in the negatively charged form. Cholesterol is a neutral material, while CPC, a cationic surfactant, is positively charged. Since PC is the major component in the formulation, it plays the key role in determining the vesicles’ surface charge. As displayed in Table 4, SL is negatively charged, so zeta potentials of F1 and F2 were found to be negative, while F3 had positive zeta potential with lower level of PC but became negatively charged when the concentration of PL increased. USPC and SSPC are neutral materials, so the zeta potentials of F1 and F2 prepared using these two PC were found to be in the range of −0.1 to 3 mV. F3 prepared using these two PCs carried a positive charge due to the positively charged CPC.

Based on the obtained data, vesicles prepared using 0.8% USPC has the highest loading of MX, with particle size less than 200 nm and uniform size distribution (PDI less than 0.3). Therefore, liposomal formulations would be prepared using 0.8% USPC in the further experiments.

#### 3.1.2. Development of Liposome Preparation Procedure

In this study, liposomes were prepared by the thin film hydration method followed by sonication. Sonication is perhaps the most widely used passive loading technique for the preparation of liposomes [23]. There are two sonicating techniques: probe and bath sonication. The effect of sonication techniques and time were studied with respect to entrapment efficiency and physical properties of the liposomal formulations. It was found that the highest entrapment efficiency was obtained by using both techniques. Initially, the liposome dispersion in a scintillation vial was placed into a bath sonicator for 1 h (Cycle 0, Table 5). Vesicles were subsequently sonicated for two cycles of 12 min using a sonication probe at continuous mode with 2 min intervals between the cycles (Cycle 1 and 2, Table 5). It was observed that prolonged sonication resulted in excessive heat in the liposomal formulations, which led to precipitation of the lipids due to the phospholipids oxidation. Therefore, the samples were kept in an ice-water bath to avoid possible oxidation. The physicochemical characteristics of F1, F2 and F3 are shown in Table 5.

The entrapment rates of MX in the vesicles were in the range of approximately 20%–80% (160–640 µg/mL). The solubility of MX in acetate buffer solution (pH 5.5) was determined to be 7 µg/mL, indicating that liposomal formulations provided substantial enhancement of MX solubility. Furthermore, the results indicated that the entrapment efficiency for transfersome incorporating CPC (84%) were much higher than that of conventional liposomes (around 20%). The intrinsic properties of the edge activator, CPC, increased the solubility of MX in vesicle bilayers. As demonstrated in Table 5, entrapment efficiency increased with increase of sonication time and plateaued at the second sonication cycle. Similar results were obtained by He et al. [33] when investigating the influence of probe-sonication on drug entrapment efficiency of ibuprofen-loaded liposomes.

As illustrated in Table 5, the vesicle sizes of different liposomal formulations were in the nano-size range of 80–130 nm with the size distribution (polydispersity index; PDI) less than 0.3, suggesting that the sonication method can prepare nano-size homogeneous vesicles. The addition of cholesterol has no impact on particle size and PDI. Transfersome had smaller vesicle sizes compared to conventional liposomes, due to the incorporation of edge activator, CPC, which can achieve higher curvature, thus resulting in decrease in vesicle size compared to conventional liposomes. It is also observed that particle size decreased with increasing sonication time and cycles. This observation agreed with the sonication study conducted by Silva et al. [34], and Nam et al. [35], in which a decrease of the particle size with the increase of the sonication time until a plateau size was obtained.

The zeta potentials of these vesicles were in a positive charge range of approximately 0.7–20 mV. Transfersome had much higher positive zeta potential compared to the conventional liposomes, which might resist aggregation and therefore provide better stability. Unlike the other two physical properties, zeta potential was not affected by the sonication time and cycles. As also indicated in the results from Section 3.1.1, addition of cholesterol has not much effect on the physiochemical properties of these liposomal vesicles. However, to keep the closest resemblance to the transfersome, F2 were used as conventional liposomes for ex vivo skin permeation study.

**Figure 4:**
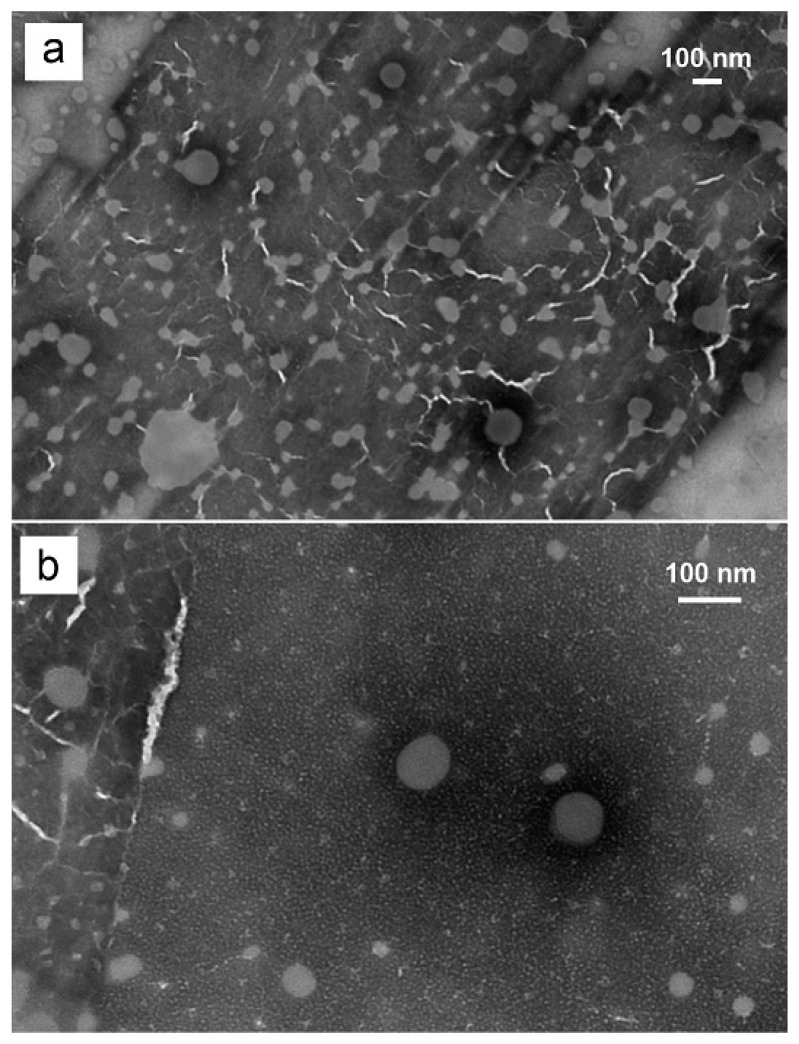
Transmission Electron Microscopy images of (**a**) F2, conventional liposome, (**b**) F3, transfersome.

To further characterize these two vesicle systems, a TEM study was conducted and Figure 4 shows a spherical shape for both formulations.

### 3.2. Microemulsions

#### 3.2.1. Pseudoternary Phase Diagrams

The surfactant applied in the preliminary phase studies was selected based on the active ingredient solubility. According to Yuan et al. [36], among different sorbitan esters, Tween^®^ 85 reveals the best properties in terms of solubilizing MX. For the oil phase, relatively polar components were tested, in order to provide the best water solubilization capacity [37].

**Figure 5:**
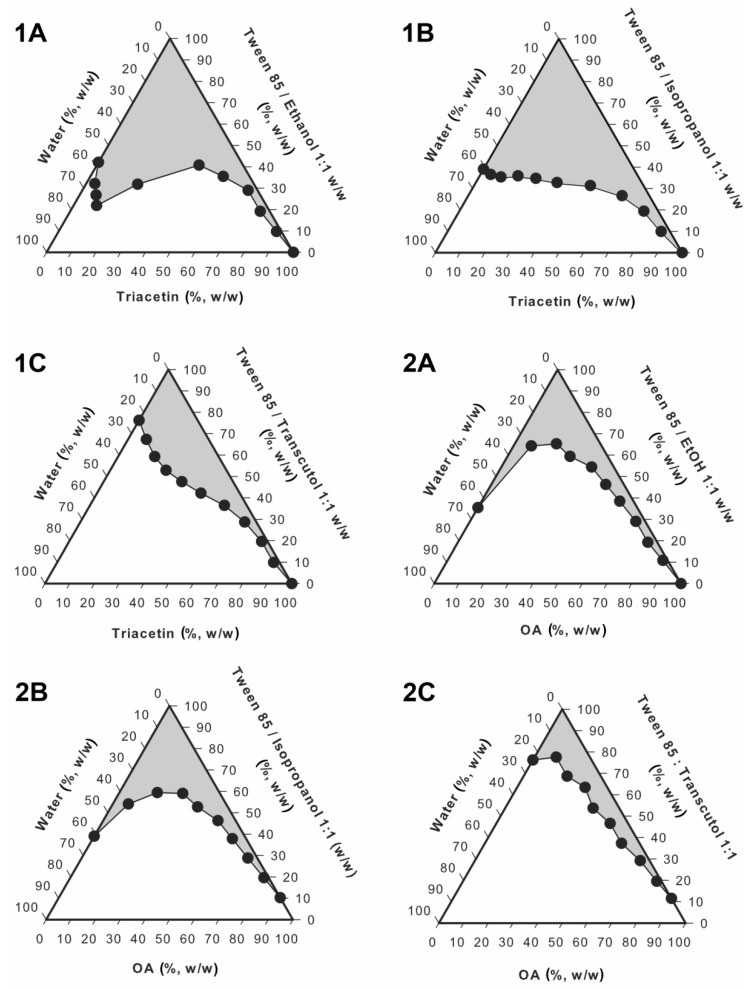
Pseudoternary phase diagrams obtained for the systems with triacetin (**1A**–**1C**) and oleic acid (**2A**–**2C**).

Pseudoternary phase diagrams obtained with different oil phases and co-surfactants are presented in Figure 5. Gray areas correspond to transparent, monophasic liquids of low viscosities identified as microemulsions, while white ones correspond to non-transparent coarse emulsions. The monophasic areas observed for the systems with triacetin (Figure 5, 1A–1C) were larger than the corresponding ones obtained for the systems with oleic acid (Figure 5, 2A–2C). The observed differences might be explained with different polarity of the applied oil phases. The values of log P reported for triacetin and oleic acid are −0.075 [38] and 3.50 [39], respectively. Lower value in the case of triacetin indicates relatively higher polarity, which results in higher water solubilization capacity. Similar results were observed by Yang et al. [40]. On the other hand, it has been hypothesized that low molecular weight oils can partially behave as co-surfactants [41], which might also contribute to the differences observed between the systems with triacetin and oleic acid investigated in this study.

In the next step, the differences observed between the systems containing different co-surfactants were taken into consideration. In both investigated sets, a significantly smaller monophasic area was observed for the systems containing Transcutol^®^ P. In the case of the systems with ethanol and isopropanol, the results are similar. However, in the case of isopropanol, monophasic areas are slightly bigger. Therefore, for the further analyses, a system with triacetin as an oil phase and isopropyl alcohol as a co-surfactant was selected.

#### 3.2.2. Conductivity Studies

Conductivity experiments were performed to assess the regions of the occurrence of particular microemulsion types. According to numerous studies [42,43,44,45], the conductivity changes observed as a result of microemulsion dilution with polar phase reflect the microstructural changes related to the transitions from one microemulsion type to another. In the initial step of the study, the observed conductivity values are close to zero, which is related to the structure and properties of water in oil (w/o) microemulsion containing isolated water droplets. The external phase in such case reveals low polarity, which is reflected by low electrical conductivity. As a result of water addition, isolated droplets start to coalesce forming polar channels which results in the transition into bicontinuous type and is reflected by the significant increase of conductivity versus water content plot slope. With further water addition, the number of channels increases which contributes to the increase of conductivity values. Finally, the bicontinuous system transforms into oil in water (o/w) microemulsion with polar external phase and electrical conductivity reaches plateau. In some cases, only a single transition point is observed and the system transforms from w/o into o/w without any discernible region corresponding to the bicontinuous system [46,47].

**Figure 6:**
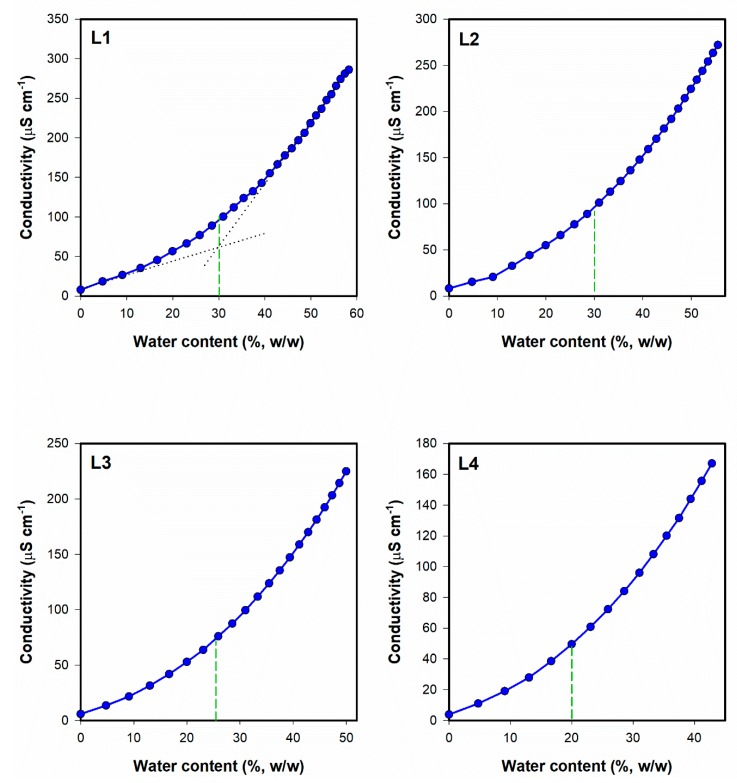
Conductivity plotted as a function of water content along the dilution lines L1–L4 for system 1B (Triacetin/Tween^®^ 85/Isopropanol). The transition points are marked with green dashed lines. The transition point estimation procedure is depicted in L1. Error bars have been omitted for clarity, and standard deviation values did not exceed 5%.

The results obtained in this study for the dilution lines L1, L2, L3 and L4 (Figure 6) indicate the presence of only one transition point corresponding to the transformation from w/o to water-continuous systems. Similar effects have been reported for other microemulsions [48,49,50].

**Figure 7:**
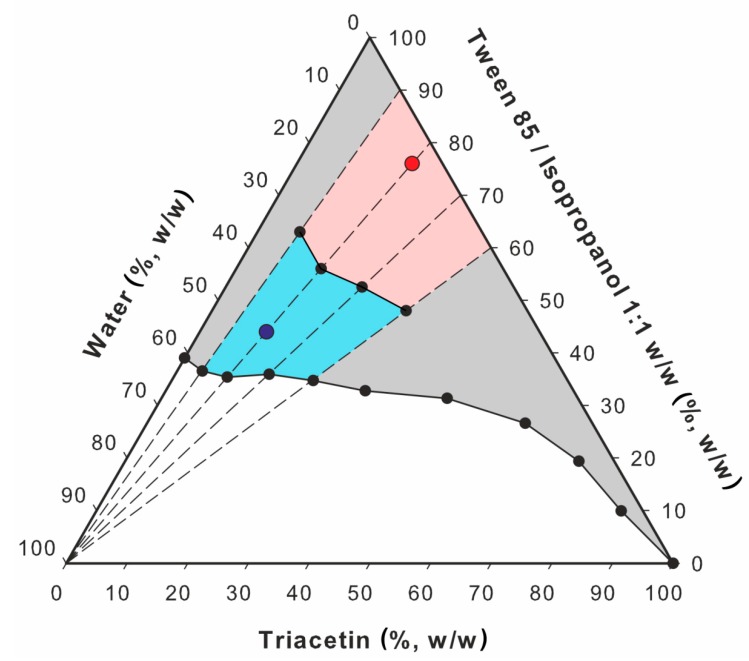
Pseudoternary phase diagram with w/o (red) and o/w (blue) microemulsion areas estimated based on the conductivity studies. Microemulsions selected for the further analyses are depicted as red and blue points (ME-1 and ME-2, respectively).

The points corresponding to the transition from w/o to water-continuous microemulsions were obtained as intersections of the extrapolated approximately linear parts of the conductivity curve, as presented in Figure 6. The transition points and the areas reflecting the occurrence of particular microemulsion types are depicted in Figure 7. Based on these findings, two microemulsion compositions from both areas were selected and applied in further experiments (Figure 7).

#### 3.2.3. Viscosity Studies

**Figure 8:**
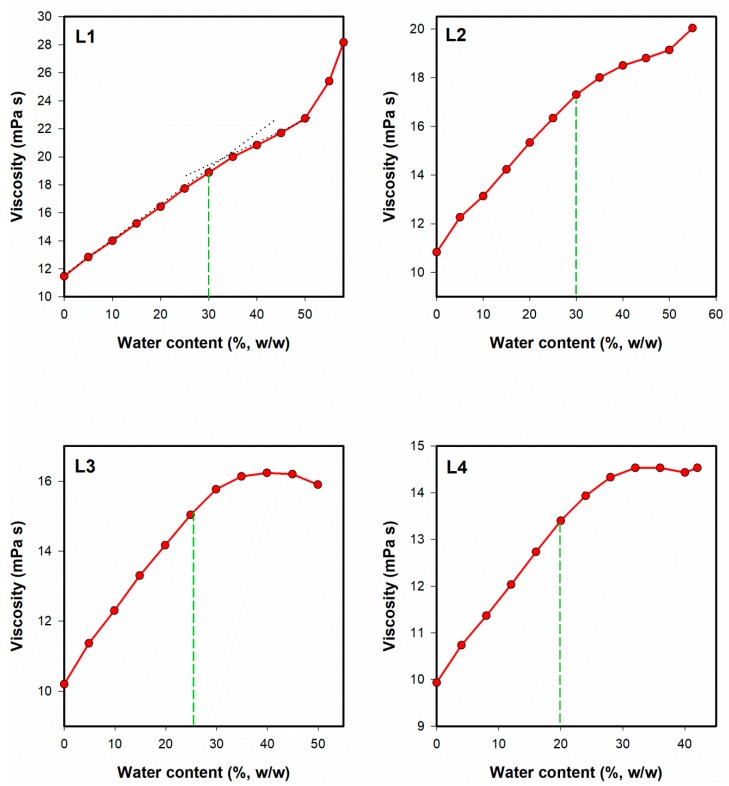
Dynamic viscosity plotted as a function of water content along the dilution lines L1–L4. The transition points estimated in conductivity studies are marked with green dashed lines. Error bars have been omitted for clarity, and standard deviation values did not exceed 5%.

In general, all investigated systems revealed Newtonian behavior, which is considered as typical for microemulsions [51], except for bicontinuous systems containing intertwining polar and non-polar domains forming an internal structure that might contribute to slightly shear-thinning behavior [52]. On the other hand, high initial viscosity of the system and highly pseudoplastic properties indicate the presence of lamellar systems which are not classified as microemulsions. It is important to notice that all analyzed systems remained liquid during the dilution and no gelation was observed. The viscosity values ranged from 10 to 28 mPa s. The plots depicting the relationship between the water content and the viscosity of microemulsions monitored along the dilution lines L1–L4 are presented in Figure 8.

Viscosity changes observed during the dilution of microemulsion can be applied for monitoring structural transitions from one microemulsion type to another [51]. In the initial phase of the experiment, w/o microemulsion is formed. The increase of viscosity in this case is related to the increased amount of the dispersed phase droplets which interact with each other. At about 25%–30% of water, the viscosity increases slower (L1 and L2, Figure 8) or reaches plateau (L3 and L4). The observed effect might be related to the structural transition leading to the formation of continuous water phase. It is noteworthy that the approximate transition points observed in viscosity curves correspond to those recorded with electrical conductivity measurements.

#### 3.2.4. Dynamic Light Scattering (DLS) Studies 

**Figure 9:**
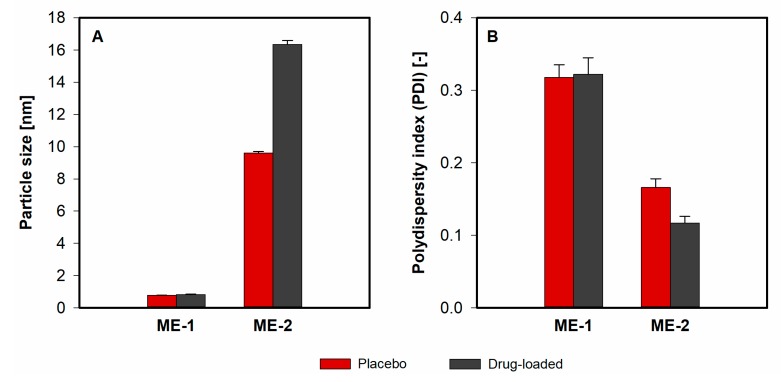
Particle size (**A**) and polydispersity index (**B**) obtained for placebo (red bars) and meloxicam-loaded (gray bars) w/o and o/w microemulsions.

**Figure 10:**
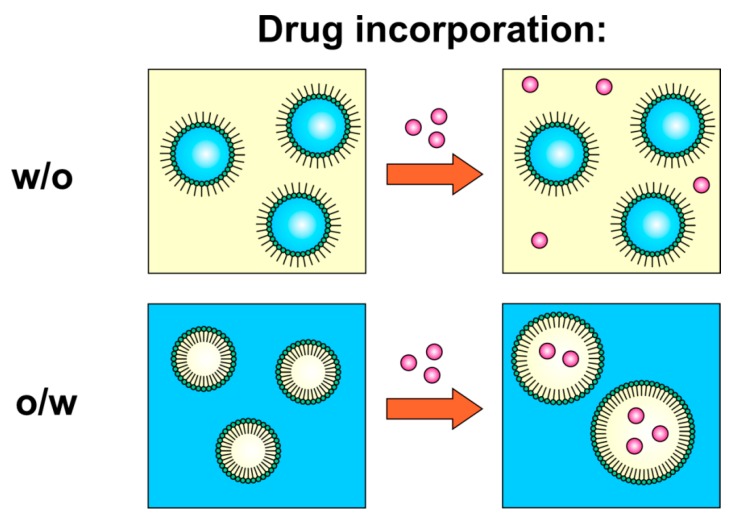
Hypothetical mechanism of drug incorporation in w/o and o/w microemulsions.

The particle size diameter and polydispersity index (PDI) values obtained for placebo and drug-loaded microemulsions are presented in Figure 9. It is noteworthy that the droplet diameter in case of water-continuous microemulsion increased with the addition of meloxicam. The obtained result might theoretically indicate the decrease of stability of the system. However, all microemulsions remained transparent during three months of storage. On the other hand, the increase of particle diameter might be related to the incorporation of an active ingredient in microemulsion droplets. In the case of an oil-continuous system, the drug remains in the external phase and its presence does not affect the particle size (Figure 10).

#### 3.2.5. Drug Permeation Studies

**Figure 11:**
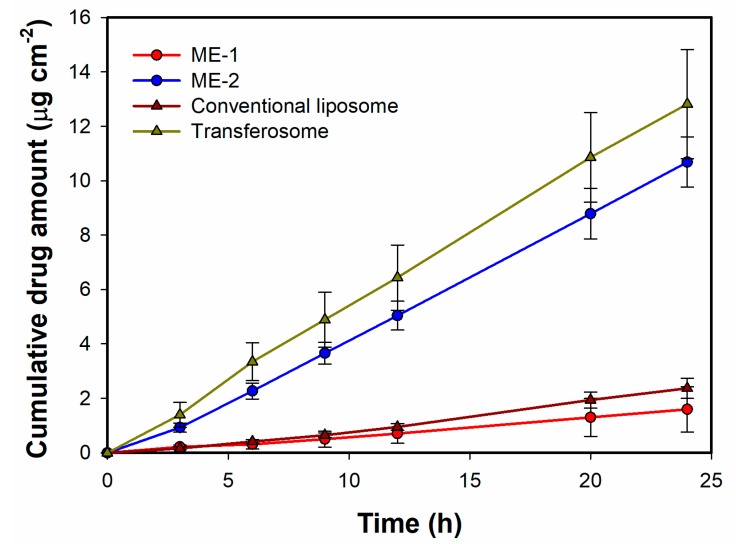
Ex vivo drug permeation profiles of MX across human cadaver skin obtained for different carriers. ME-1 and ME-2 are w/o and o/w microemulsions, respectively. Data are plotted as means ± SD (*n* = 3 for each formulation).

Ex vivo skin permeation studies were conducted with both liposome and microemulsion formulations applied to human cadaver skin using Franz diffusion cells. The results of skin permeation experiments yielded plots of cumulative drug amount versus time and are presented in Figure 11, while the values of steady state flux (J_ss_) and permeability coefficients (K_p_) are presented in Table 6. Steady state flux values were calculated as a slope of the linear plots presented in Figure 10, while permeability coefficients were calculated with the use of Equation (3) [53]. Both parameters calculated for liposomal formulations clearly indicate that transfersomes reveal a greater ability to penetrate the skin compared to the classical non-deformable liposomes, suggesting that the deformable liposomes greatly enhanced the permeation of MX compared to the rigid vesicles. The mechanisms underlying the differences observed in this study have been described in the literature related to deformable vesicles [54,55,56,57]. The most important structural feature of transfersomes is the presence of surfactants acting as edge activators and destabilizing lipid bilayers. As a result, the modified vesicles are more flexible and susceptible to deformation than the conventional ones, which allows for more efficient penetration through the pores present in the skin. Another important factor in the permeation enhancement is osmotic gradient, which acts as a driving force pushing transfersomes from the relatively dehydrated skin surface into the deeper layers of skin. It is worth mentioning that the osmotic effect was reported as crucial in non-occlusive conditions [58] while in this study, all samples placed in Franz diffusion cells were protected from water evaporation. Therefore, it may be hypothesized that the differences observed between conventional and deformable vesicles could be even higher in a non-occlusive environment.

(3) $$K_{p}=\frac{J_{ss}}{C_{d}}$$

where:

*J_ss_*—steady state flux (μg cm^−2^ h^−1^), and
*C_d_*—concentration of MX in the donor compartment (µg mL^−1^).

The drug permeation results obtained for two different microemulsions investigated in this study indicate that the effectiveness of the carrier depends on the type of microemulsion or water content in the system. In the case of o/w microemulsion, the steady state flux value is significantly higher than that in the w/o system. Similar effects have been reported by Zhang and Michniak-Kohn [59]. It was shown that the increase of water content in the system resulted in the increased drug permeation, which was more pronounced for lipophilic drugs when compared to a hydrophilic one. However, in the case of active ingredients revealing low solubility in water, the described effect might be related to the increased thermodynamic activity of the drugs in water-rich systems.

Similar tendencies were described in the study focusing on hydrophilic caffeine [60]. The highest flux values were observed for o/w microemulsions, while the lowest ones were recorded for oil-continuous systems. However, the authors indicated lower permeation differences observed between the particular microemulsion types than in the case of less polar actives reported in the literature. The statistically significant differences obtained in this study for two structurally different microemulsion systems can also be related to the hydrophobic character of the applied drug.

**Figure 12:**
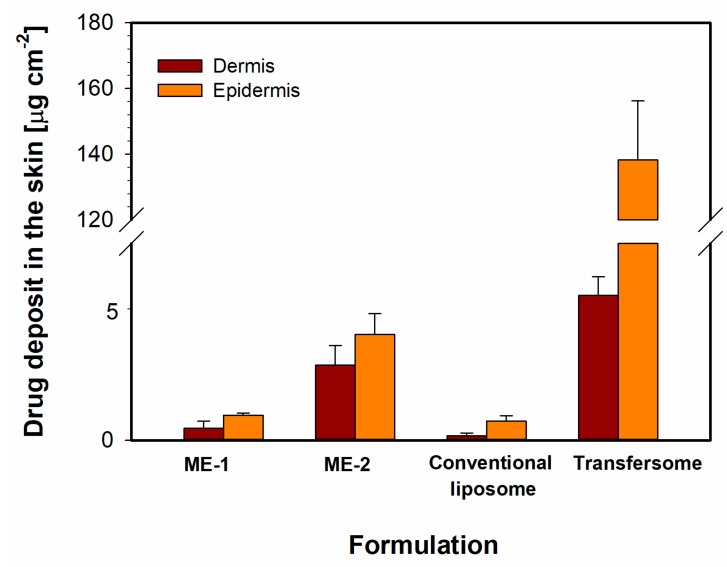
Amounts of meloxicam deposited in the skin layers after 24 h of experiment. Data are plotted as means ± SD (*n* = 3 for each formulation).

The comparison of the drug flux values calculated for different microemulsions and liposomal formulations revealed statistically significant results between the investigated formulations. According to Figure 11, conventional liposomes displayed similar properties as w/o microemulsion, while for transfersomes and o/w microemulsion, higher flux values were obtained. Comparing the potential therapeutic utility of the investigated formulations, it may be expected that the latter two will perform better. A similar tendency was also observed for the amounts of the drug deposited in the skin (Figure 12). The highest concentration of meloxicam in both skin layers was recorded for transfersomes, which indicated the highest ability to overcome *stratum corneum* and also explained the highest concentration in the receptor medium. The amounts observed for o/w microemulsion were lower, even though the drug flux was very similar. This may indicate a higher tendency to penetrate deeper with apparently lower affinity to the dermis and epidermis. The observed effect can be explained with the composition of both systems. Transfersomes contain naturally derived phospholipids which display high biocompatibility and high affinity to skin structures. Therefore, it might be expected that higher amounts of the drug incorporated in the vesicles will be retained in the skin. On the other hand, conventional liposomes containing the same phospholipids reveal lower elasticity and ability to deform, which decreases their ability to overcome the skin barriers and stay in the skin structures. These results suggested that the transfersomes containing cationic surfactant may affect the lipids of SC and therefore produce an enhancing effect in terms of dermal drug delivery.

In the case of microemulsions, it might be hypothesized that the presence of surfactants and co-surfactants contributed to the increased ability to penetrate through the *stratum corneum* into the deeper layers of the skin without binding to them, as was observed in transfersomes. However, the tendency was not the same for different types of microemulsions, which indicated the possible impact of the structural features and thermodynamic activity of the incorporated drug. It is noteworthy that the literature reports showing the comparison between microemulsions and liposomal formulations are quite scarce. According to El-Badry et al. [61], microemulsions with two different co-surfactants revealed higher drug flux than liposomal formulations containing croconazole, a poorly water-soluble antimycotic drug. Nevertheless, the experiment was conducted with the use of an animal skin model, which might provide different results when compared to human cadaver skin. Moreover, the efficacy of microemulsion is dependent on its composition. The study presented by Yuan et al. [36] investigating meloxicam-loaded microemulsions showed significantly higher drug flux values. However, isopropyl myristate applied as an oil phase is less polar than triacetin applied in our study, which might potentially contribute to the observed differences.

Taking into consideration the permeation coefficients calculated for all investigated formulations, it may be assumed that the observed differences are partially related to different drug content occurring mostly as a result of different encapsulation efficiency in liposomes. The highest value was obtained for transfersomes, while conventional liposomes and water-continuous microemulsion revealed similar properties. The lowest value of permeation coefficient was observed for oil-continuous microemulsion. The comparison made between two different types of vesicles confirmed the results described in the literature [62,63,64]. The available studies show that transfersomes reveal better properties in terms of dermal drug delivery, which is related to higher deformability allowing for better penetration into deeper skin layers. Moreover, because of the presence of cationic surfactant, transfersomes may disrupt the organization of lipids in the *stratum corneum*. Similar results, indicating that transfersomes also had higher tendency to be retained in skin when compared to conventional liposomes, were presented by Alvi et al. [63].

Considering advantages and disadvantages of the investigated dermal delivery systems, it can be assumed that transfersomes reveal better properties as potential drug carriers, efficiently enhancing the absorption of the active ingredient. Another important feature of phospholipid-based vesicles is their high compatibility with the skin and low irritancy, which is particularly important in longer therapies requiring multiple applications. According to Mahrhauser et al. [65], liposomes and multiple emulsions had no negative effects on the skin, while microemulsion-based formulation temporarily increased transepidermal water loss, which resulted in dehydration. The observed side effects related to microemulsion administration might be related to high surfactants and co-surfactants content. On the other hand, microemulsions are thermodynamically stable dispersions, which means that the formation process is spontaneous and does not require high amounts of energy and complicated multi-step technological processes. Therefore, several technological difficulties, such as non-uniformity, instability or high variability of the final product, can be avoided.

## 4. Conclusions

In the study, conventional liposomes and deformable transfersomes were obtained and compared to two different types of microemulsions as potential dermal delivery carriers for meloxicam, a non-steroidal anti-inflammatory drug. The performed studies allowed for optimization of the preparation method and composition of the investigated systems. When comparing the w/o and o/w microemulsion performance with the use of an ex vivo model involving human cadaver skin, the highest flux and permeation values were obtained for transfersomes, indicating these drug carriers as the most promising in terms of topical drug delivery.

## Figures and Tables

**Table 1:** Composition of the samples investigated in phase studies.

No	Oil Phase	S_mix_		Water Phase
		Surfactant	Co-Surfactant	
1A	Triacetin	Tween^®^ 85	Ethanol	Water
1B			Isopropanol	
1C			Transcutol^®^ P	
2A	Oleic acid		Ethanol	
2B			Isopropanol	
2C			Transcutol^®^ P	

**Table 2:** The composition of microemulsions used for Meloxicam (MX) incorporation.

Component (%, *w/w*)	Sample	
	ME-1	ME-2
Triacetin	19.0	11.0
Tween 85	38.0	22.0
Isopropyl alcohol	38.0	22.0
Water	5.0	45.0

**Table 3:** The composition and type of the vesicles investigated in the study.

Formulation ID	Description	Wt (mg)/100 mL			
		PC ^1^	MX ^2^ (0.08%)	Cholesterol ^3^ (0.04%)	CPC ^4^ (0.10%)
F1	Conventional Liposome	400/800/1200	80	-	-
F2	Conventional Liposome	400/800/1200	80	40	-
F3	Transfersome	400/800/1200	80	40	100

^1^ Phosphatidylcholine, ^2^ Meloxicam, ^3^ Cholesterol, ^4^ Cetylpyridinium chloride.

**Table 4:** Physicochemical properties of the obtained vesicles (*n* = 3); a: soybean lecithin, b: unsaturated soybean phosphatidylcholine, c: saturated soybean phosphatidylcholine, d: Polydispersity Index (PDI).

**SL ^a^**	**0.4%**			**0.8%**			**1.2%**		
**Formulation ID**	**Ave Diameter (nm)**	**PDI**	**Zeta Potential (mV)**	**Ave Diameter (nm)**	**PDI**	**Zeta Potential (mV)**	**Ave Diameter (nm)**	**PDI**	**Zeta Potential (mV)**
**F1**	113.4 ± 2.0	0.173 ± 0.010	−11.6 ± 0.3	119.6 ± 2.6	0.185 ± 0.011	−25.7 ± 0.5	133.7 ± 0.2	0.191 ± 0.013	−23.4 ± 1.3
**F2**	130.5 ± 1.8	0.212 ± 0.010	−26.5 ± 0.8	125.7 ± 2.5	0.178 ± 0.019	−23.0 ± 0.5	133.9 ± 2.0	0.192 ± 0.019	−23.7 ± 0.5
**F3**	128.0 ± 2.8	0.219 ± 0.014	6.7 ± 0.5	155.4 ± 11.2	0.215 ± 0.027	−10.7 ± 0.6	172.1 ± 1.8	0.150 ± 0.014	−9.6 ± 0.4
**USPC ^b^**	**0.4%**			**0.8%**			**1.2%**		
**Formulation ID**	**Ave Diameter (nm)**	**PDI**	**Zeta Potential (mV)**	**Ave Diameter (nm)**	**PDI**	**Zeta Potential (mV)**	**Ave Diameter (nm)**	**PDI**	**Zeta Potential (mV)**
**F1**	125.0 ± 3.0	0.226 ± 0.020	0.2 ± 0.5	126.2 ± 0.9	0.259 ± 0.024	0.0 ± 1.5	120.5 ± 0.4	0.186 ± 0.006	−1.3 ± 1.7
**F2**	114.2 ± 3.9	0.256 ± 0.008	-0.1 ± 0.8	125.7 ± 4.9	0.274 ± 0.052	−0.2 ± 0.6	121.7 ± 0.9	0.205 ± 0.017	−0.3 ± 0.3
**F3**	103.0 ± 0.3	0.254 ± 0.009	27.1 ± 0.8	136.7 ± 5.7	0.185 ± 0.019	20.0 ± 0.6	174.0 ± 3.1	0.408 ± 0.021	16.9 ± 0.1
**SSPC ^c^**	**0.4%**			**0.8%**			**1.2%**		
**Formulation ID**	**Ave Diameter (nm)**	**PDI**	**Zeta Potential (mV)**	**Ave Diameter (nm)**	**PDI**	**Zeta Potential (mV)**	**Ave Diameter (nm)**	**PDI**	**Zeta Potential (mV)**
**F1**	2347 ± 325.9	0.859 ± 0.126	1.1 ± 0.3	445.5 ± 3.6	1.000 ± 0.000	0.7 ± 0.3	270.4 ± 20.7	0.947 ± 0.091	1.0 ± 0.3
**F2**	168.5 ± 4.4	0.308 ± 0.021	2.7 ± 0.8	190.1 ± 1.4	0.430 ± 0.026	3.4 ± 1.2	160.6 ± 5.2	0.510 ± 0.023	1.1 ± 0.3
**F3**	445.1 ± 11.7	0.318 ± 0.041	40.7 ± 1.9	199.0 ± 0.6	0.351 ± 0.062	34.3 ± 1.5	178.8 ± 3.6	0.405 ± 0.017	27.8 ± 0.5

**Table 5:** Physicochemical properties of the samples F1, F2 and F3 subjected to the sonication procedure (*n* = 3).

Formulation ID	Cycle No	%Entrapment Efficiency	Average Diameter (nm)	PDI *	Zeta Potential (mV)
F1	0	17.60 ± 0.64	126.4 ± 2.0	0.221 ± 0.013	0.78 ± 0.27
	1	20.96 ± 0.47	121.3 ± 0.9	0.243 ± 0.005	0.82 ± 0.23
	2	21.91 ± 0.52	96.1 ± 1.3	0.283 ± 0.008	0.73 ± 0.34
F2	0	19.83 ± 0.26	129.7 ± 5.3	0.235 ± 0.003	1.17 ± 0.26
	1	20.17 ± 0.40	111.4 ± 0.2	0.257 ± 0.008	1.01 ± 0.36
	2	22.13 ± 0.35	100.6 ± 1.1	0.237 ± 0.010	0.89 ± 0.54
F3	0	74.26 ± 2.83	96.72 ± 1.5	0.258 ± 0.008	20.8 ± 0.5
	1	83.57 ± 1.89	85.36 ± 0.8	0.266 ± 0.004	19.9 ± 1.2
	2	83.59 ± 0.73	73.46 ± 1.3	0.253 ± 0.007	21.4 ± 0.6

* PDI: Polydispersity Index.

**Table 6:** Steady state flux (J_ss_) and permeation coefficients (K_p_) obtained for the investigated formulations. ME-1 and ME-2 are w/o and o/w microemulsions, respectively. Data are presented as means ± SD (*n* = 3 for each formulation).

Formulation	J_ss_ (μg cm^−2^ h^−1^)	Concentration(µg mL^−1^)	K_p_ (cm h^−1^)
Conventional liposome	0.11 ± 0.02	177.04	(60.75 ± 11.94) × 10^−5^
Transfersomes	0.54 ± 0.08	668.75	(80.82 ± 12.28) × 10^−5^
ME-1	0.07 ± 0.04	735.50	(9.59 ± 4.84) × 10^−5^
ME-2	0.46 ± 0.04	768.71	(60.50 ± 5.12) × 10^−5^
